# Rheb1 protects against cisplatin-induced tubular cell death and acute kidney injury via maintaining mitochondrial homeostasis

**DOI:** 10.1038/s41419-020-2539-4

**Published:** 2020-05-13

**Authors:** Qingmiao Lu, Mingjie Wang, Yuan Gui, Qing Hou, Mengru Gu, Yan Liang, Bo Xiao, Allan Zijian Zhao, Chunsun Dai

**Affiliations:** 1 Center for Kidney Disease, 2nd Affiliated Hospital, Nanjing Medical University, 262 North Zhongshan Road, Nanjing, Jiangsu China; 2 Department of Biology, Southern University of Science and Technology, 518000 Shenzhen, P.R. China; 3 Institute of Biomedical and Pharmaceutical Sciences, Guangdong University of Technology, 510515 Guangzhou, P.R. China

**Keywords:** Cell death, Acute kidney injury

## Abstract

Ras homolog enriched in brain (Rheb1), a small GTPase, plays a crucial role in regulating cell growth, differentiation, and survival. However, the role and mechanisms for Rheb1 in tubular cell survival and acute kidney injury (AKI) remain unexplored. Here we found that Rheb1 signaling was activated in kidney tubule of AKI patients and cisplatin-treated mice. A mouse model of tubule-specific deletion of Rheb1 (Tubule-Rheb1^−/−^) was generated. Compared to control littermates, Tubule-Rheb1^−/−^ mice were phenotypically normal within 2 months after birth but developed more severe kidney dysfunction, tubular cell death including apoptosis, necroptosis and ferroptosis, mitochondrial defect and less PGC-1*α* expression after cisplatin injection. In primary cultured tubular cells, Rheb1 ablation exacerbated cisplatin-induced cell death and mitochondrial defect. Furthermore, haploinsufficiency for Tsc1 in tubular cells led to Rheb1 activation and mitigated cisplatin-induced cell death, mitochondrial defect and AKI. Together, this study uncovers that Rheb1 may protect against cisplatin-induced tubular cell death and AKI through maintaining mitochondrial homeostasis.

## Introduction

Acute kidney injury (AKI), a clinical syndrome characterized by an abrupt loss of kidney function within hours, is a worldwide health problem with high mortality^1^. Although tremendous effort has been made by the nephrologist during the past decades, the therapeutic strategy for AKI patients is mainly supportive in nature up to date^2^. Therefore, deciphering the underlying mechanisms of AKI and pursuing efficient strategy for preventing and treating AKI in patients is necessary.

The pathogenic factors for AKI in patients are mainly comprised by ischemia, renal toxic reagents, and sepsis^3^. Although tubular cell injury and death, peritubular endothelial dysfunction and inflammatory cell infiltration all contribute to the initiation and progression of AKI^4,5^, tubular cell injury and death is considered to be the pivotal one^6,7^. Under physiologic condition, tubular cells demand high energy supplied by mitochondria to support its normal function and survival and mitochondrial defect may lead to tubular cell injury and death^8,9^. In this regard, deciphering the mechanisms for maintaining mitochondrial homeostasis in tubular cells is essential for clarifying the pathogenesis of AKI.

In mammalian cells, there are two identified genes for Ras homolog enriched in brain (Rheb): *Rheb1* (called *Rheb*) and *Rheb2* (called *Rhebl1*). The products of *Rheb1* and *Rheb2* genes share 54% identity and 74% similarity. Of them, Rheb1 is ubiquitously expressed while Rheb2 is mainly expressed in brain^10^. Similar to the other small GTPases, Rheb1 protein has two forms of existence: inactive GDP-bound and active GTP-bound states, and may be deactivated by Tsc1/Tsc2 complex^11,12^. Rheb1 is localized at cellular compartments, such as mitochondria, lysosome, and peroxisome, and functions as a molecular switch in various cellular functions, including cell growth, survival, and death^13–18^. Tian et al. revealed that Rheb1 may repress cell apoptosis and promote cell proliferation in colorectal cancer cells^19^. Cao et al. demonstrated that ablation of Rheb1 in cardiomyocytes induces heart growth impairment, aberrant metabolism-related gene expression and cardiomyocyte apoptosis^20^. Our previous studies revealed that activation of kidney fibroblast Rheb1 may induce kidney interstitial fibrosis^21^, while ablation of fibroblast Rheb1 promotes tubular cell death and kidney ischemia/reperfusion injury (IRI) in mice^22^. However, the role and mechanisms for tubular cell Rheb1 in regulating tubular cell survival and AKI remain unknown yet.

In this study, we found that Rheb1 signaling was activated in the kidney tubule of AKI patients and mice with cisplatin-induced AKI. Specific ablation of Rheb1 in tubule deteriorated cisplatin-induced tubular cell mitochondrial defect, cell death and AKI. In addition, haploinsufficiency of Tsc1 in tubular cells led to Rheb1 signaling activation and prevented cisplatin-induced mitochondrial defect, tubular cell death and AKI in both mouse model and primary cultured tubular cells. Our results demonstrate that tubular Rheb1 protects against tubular cell death and AKI through maintaining mitochondrial homeostasis.

## Methods

### Mice and animal models

All animals were maintained in Specific Pathogen-Free (SPF) Laboratory Animal Center of Nanjing Medical University according to the guidelines of the Institutional Animal Care and Use Committee from Nanjing Medical University. Homozygous Rheb1 floxed mice (C57BL/6J background) were kindly provided by Dr. Xiao^23^. Tsc1 floxed mice were ordered from Jackson Laboratory (cat: 005680, Jackson Labs, Bar Harbor, ME). The Ksp1.3/Cre transgenic mice were ordered from Jackson lab (cat: 012237, C57BL/6J background). Rheb1^fl/fl^ mice were crossed to Ksp-Cre mice to generate offspring with specific deletion of Rheb1 in tubular epithelial cells (Tubule-Rheb^−/−^, genotype: Cre^+/−^, Rheb1^fl/fl^). The same gender mice genotyping Cre^−/−^, Rheb^fl/fl^ from the same litters were considered as control littermates. Tsc1^fl/fl^ mice were crossbred with Ksp-Cre mice to generate mice with tubular cell haploinsufficiency of Tsc1 gene (Tubule-Tsc1^+/−^, genotype: Cre^+/−^, Tsc1^fl/wt^). The same gender mice genotyping Cre^−/−^, Tsc1^fl/fl^ from the same litters were considered as control littermates. Genotyping was performed by PCR using DNA extracted from mouse tails. The primers used for genotyping were as follows: Cre transgene, sense: 5′-CTGATTTCGACCAGGTTCGT-3′ and antisense: 5′-ATTCTCCCACCGTCAGTACG-3′; Rheb1 gene, sense: 5′-GCCCAGAACATCTGTTCCAT-3′ and anti-sense: 5′-GGTACCCACAACCTGACACC-3′; Tsc1 gene, sense: 5′-GTCACGACCGTA GGAGAAGC-3′ and anti-sense: 5′-GAATCAACCCCACAGAGCAT-3′. All animals were born normal with the expected Mendelian frequency. To induce AKI in mice, Tubule-Rheb1^−/−^, Tubule-Tsc1^+/−^ and their control littermates aged between 8 and 10 weeks were injected with a single dose of 20 mg/kg cisplatin (cat: P4394, Sigma-Aldrich, St. Louis, MO) intraperitoneally. Mice were sacrificed at day 1, 2 and 3 after cisplatin administration, and mice in AKI models died before being sacrificed were excluded. Blood and kidney samples were harvested for further analysis.

### Cell culture and treatment

Tubular epithelial cells isolated from kidneys of Rheb1^fl/fl^ and Tsc1^fl/wt^ mice were cultured in Dulbecco’s modified Eagle’smedium-F12 medium supplemented with 10% fetal bovine serum (Invitrogen, Grand Island, NY) and infected with adenovirus carrying Cre recombinase gene to generate tubular cell with Rheb1 ablation and haploinsufficiency of Tsc1, respectively. Tubular cells were incubated with 25 mg/ml cisplatin (cat: P4394, Sigma-Aldrich, St. Louis, MO) in culture medium for 12 or 24 h to induce cell death.

### Statistical analyses

Data were expressed as mean ± s.e.m. Western blot analysis was completed by scanning and analyzing the intensity of hybridization signals by using the NIH Image program. Statistical analysis of data was performed using the Graphpad Prism 6 (GraphPad Software, San Diego, CA). Comparison between groups was made using one-way analysis of variance, followed by the Student–Newman–Keuls test. *P* < 0.05 was considered as statistically significant.

## Results

### Rheb1 signaling is activated in kidney tubular cells from AKI patients and mice with cisplatin-induced AKI

**Fig. 1:**
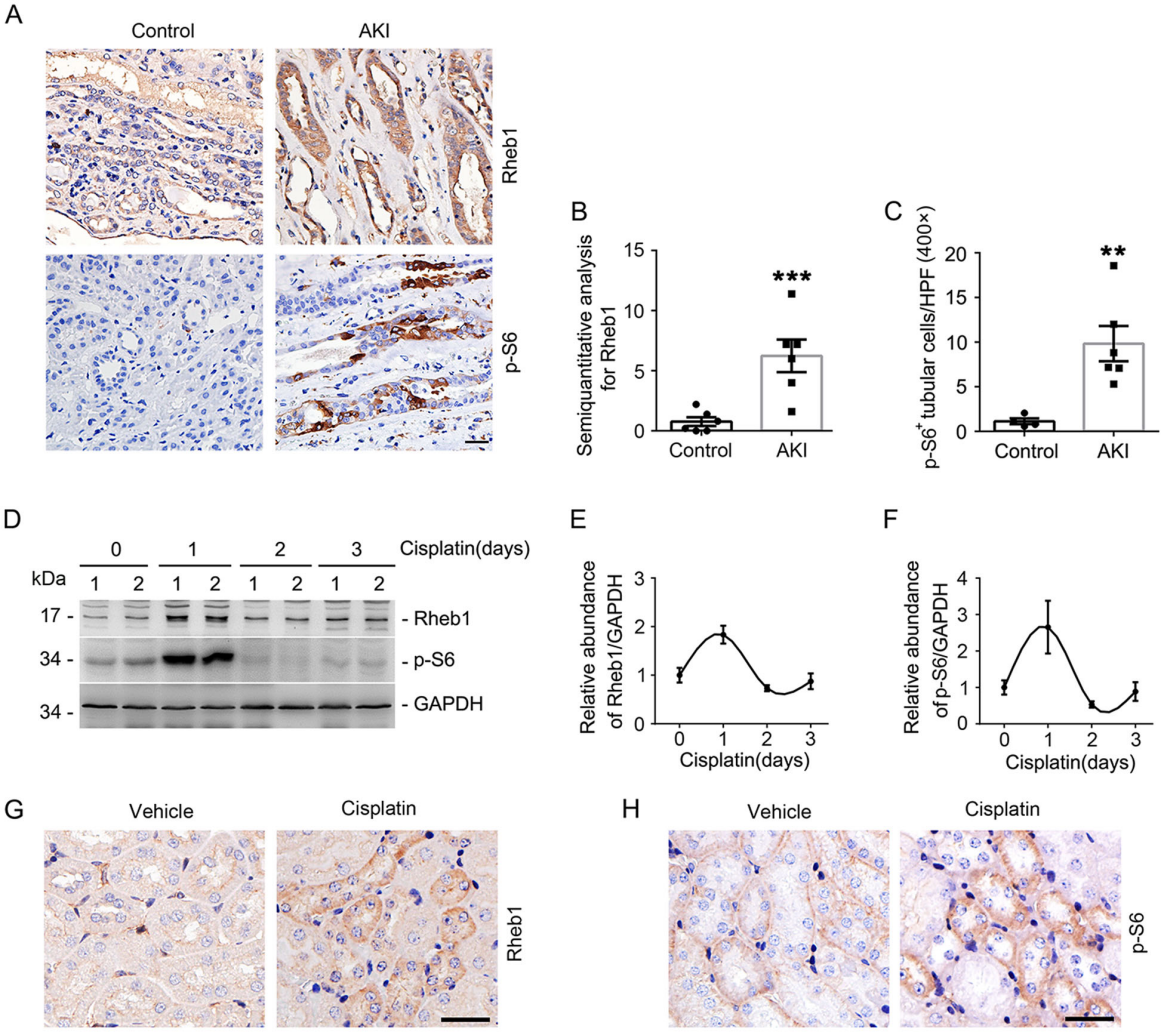
Activation of Rheb1 signaling in kidney tubule from AKI patients and mice with cisplatin-induced AKI. **a** Representative immunohistochemical staining images showing the induction of Rheb1 and p-S6 in kidney tubule from AKI patients. Scale bar = 20 μm. **b**, **c** Quantitative analyses of Rheb1 (**b**) and p-S6-staining (**c**) positive tubular cells in renal biopsies. ***P < 0.001, **P < 0.01 vs. Control, *n* = 6. **d**–**f** Western blot assay (D) and quantitative analyses (**e**, **f**) showing the induction of Rheb1 and p-S6 in kidneys from mice after cisplatin injection, *n* = 4 for each point. Male CD1 mice were intraperitoneally injected with cisplatin at 20 mg/kg and kidney tissues were harvested at day 1, 2, and 3 thereafter. **g**, **h** Representative immunohistochemical staining images showing the induction of Rheb1 and p-S6 in kidney tubule from mice at day 1 after cisplatin injection. Scale bar = 20 μm.

To investigate the role for Rheb1 signaling in AKI, we first performed immunohistochemical staining for Rheb1 and one of its downstream signaling molecules p-S6 on renal biopsies from AKI patients. The adjacent kidney tissues of kidney tumor from patients were used as controls. The results showed that weak Rheb1 and little p-S6 were detected in control kidney tissues, while in kidney tubule of AKI patients, both Rheb1 and p-S6 abundance was significantly increased (Fig. 1a–c).

We next examined the abundance of Rheb1 and p-S6 in mouse kidneys with cisplatin-induced AKI. Western blot analysis showed that both Rheb1 and p-S6 abundance was largely increased in the kidneys at day 1, but returned to baseline at day 2 and 3 after cisplatin injection (Fig. 1d–f). Immunohistochemistry staining showed that the induction of Rheb1 and p-S6 were mainly localized in tubular cells (Fig. 1g, h). Together, these results suggest that Rheb1 signaling is activated in kidney tubule from both patients and animal models with AKI.

### Specific deletion of Rheb1 in tubular cells aggravates cisplatin-induced AKI in mice

**Fig. 2:**
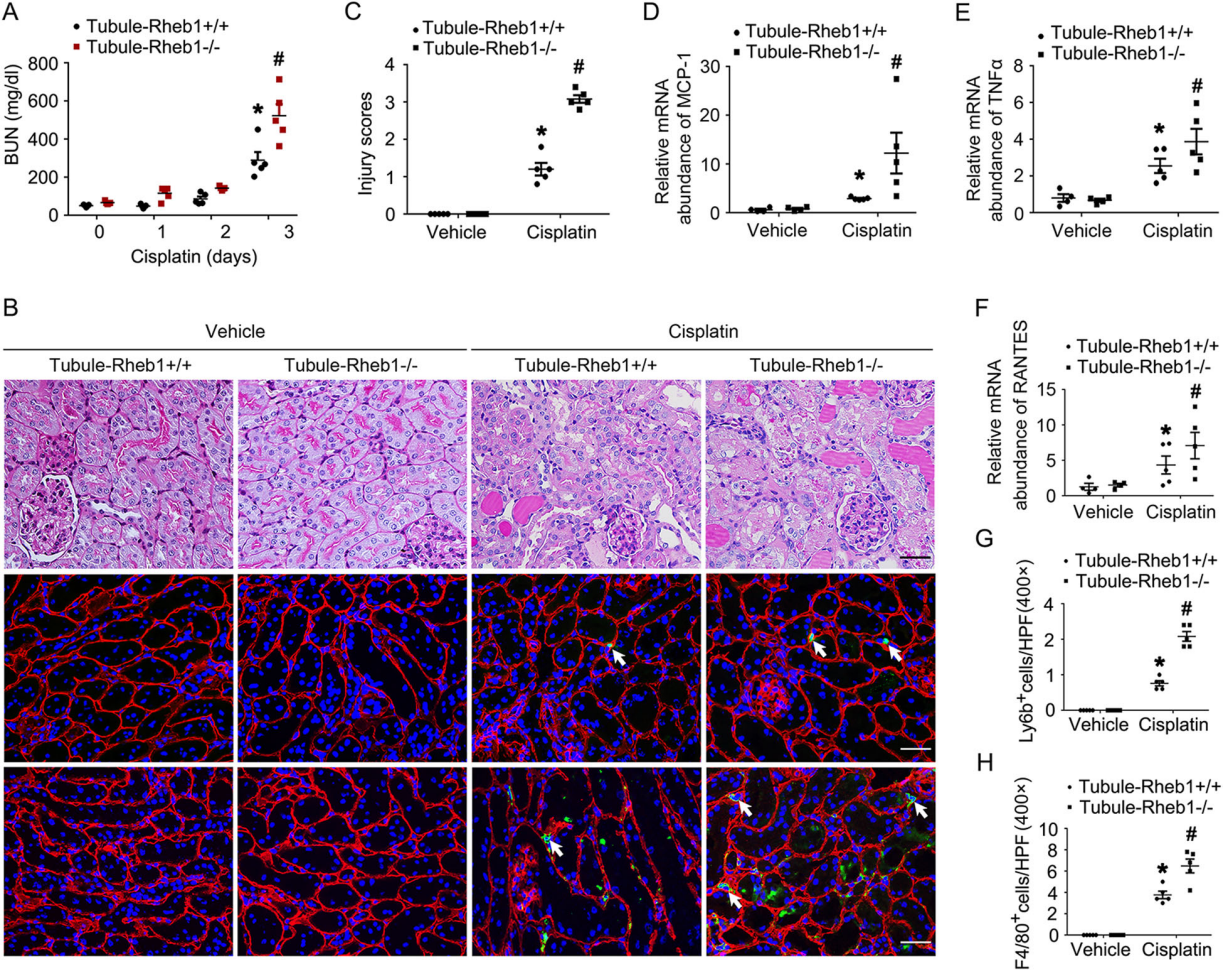
Mice with tubular cell-specific deletion of Rheb1 are more susceptible to cisplatin-induced AKI. **a** The graph showing blood urea nitrogen (BUN) level in Tubule-Rheb1^+/+^ and Tubule-Rheb1^−/−^ mice at day 0, 1, 2, and 3 after cisplatin injection. *P < 0.05 vs. Vehicle control, #*P* < 0.05 vs. Tubule-Rheb1^+/+^ mice injected with cisplatin, *n* = 5. **b** Kidney histology as shown by periodic acid–Schiff (PAS) staining. Representative immunofluorescent staining images for Ly6b and F4/80. The sections were co-stained with anti-Laminin to outline the tubular area. White arrows indicate staining positive inflammatory cells. Scale bar = 20 μm. **c** Injury scores. *P < 0.05 vs. Vehicle control, *n* = 5, #*P* < 0.05 vs. Tubule-Rheb1^+/+^ mice injected with cisplatin, *n* = 5. Each dot represents the average of five HPFs from each mouse. **d**–**f** Real-time PCR analysis showing the mRNA abundance for MCP-1, TNF*α* and RANTES. **g**, **h** Quantitative analyses of Ly6b-staining positive and F4/80-staining positive cells among groups as indicated. *P < 0.05 vs. Vehicle control, *n* = 5, #*P* < 0.05 vs. Tubule-Rheb1^+/+^ mice injected with cisplatin, *n* = 5. Each dot represents the average of five HPFs from each mouse.

To elucidate the role of tubular cell Rheb1 induction in AKI, we generated conditional knockout mice in which Rheb1 gene was deleted in tubular cells by using the Cre-LoxP system (Supplementary Fig. 1A). By cross breeding of Rheb1 floxed mice and Ksp-Cre transgenic mice, we obtained knockout mice with genotype Ksp-Cre^+/−^, Rheb1^fl/fl^ (Tubule-Rheb1^−/−^, Supplementary Fig. 1B, lane 2). Littermates with genotype Ksp-Cre^−/−^, Rheb1^fl/fl^ were used as controls (Tubule-Rheb1^+/+^, Supplementary Fig. 1B, lane 1). Western blot analysis demonstrated the reduction of Rheb1 protein in the kidneys (Supplementary Fig. 1C). To further examine the Rheb1 expression within the different tubular segments, we co-stained the kidney tissues with antibody against Rheb1 and FITC-conjugated proximal tubular cell marker PHA-E or FITC-conjugated distal tubular cell marker PNA, respectively. The results showed that Rheb1 expression was reduced in both proximal tubular cells and distal tubular cells (Supplementary Fig. 1D). Within 2 months after birth, there was no significant difference as to body weight, kidney weight, kidney/body weight ratio, urinary glucose, BUN, urinary NAG level, and kidney histology between the knockouts and control littermates (Supplementary Fig. 1E–K). Mice were injected with cisplatin to induce AKI. As shown in Fig. 2a, much more BUN level was observed in the knockouts compared to control littermates at day 3 after cisplatin injection. The loss of brush border, enlargement of tubular lumen and tubular cell loss were more severe in Tubule-Rheb1^−/−^ mice compared to their control littermates (Fig. 2b, c). In addition, kidney inflammatory response was observed. The results of real time PCR assay showed Monocyte chemotactic protein-1 (MCP1), the regulated on activation normal T cell expressed and secreted (RANTES) and TNF*α* mRNA abundance were markedly increased in control kidneys after cisplatin injection, which was more severe in Tubule-Rheb1^−/−^ mice after cisplatin injection (Fig. 2d–f). Similarly, inflammatory cell infiltration was more severe in Tubule-Rheb1^−/−^ mice compared to their control littermates after cisplatin injection (Fig. 2b, g, h). Together, these results indicate that ablation of tubular cell Rheb1 aggravates cisplatin-induced tubular cell injury and AKI in mice.

### Specific deletion of Rheb1 in tubular cells promotes cisplatin-induced tubular cell death in mice

**Fig. 3:**
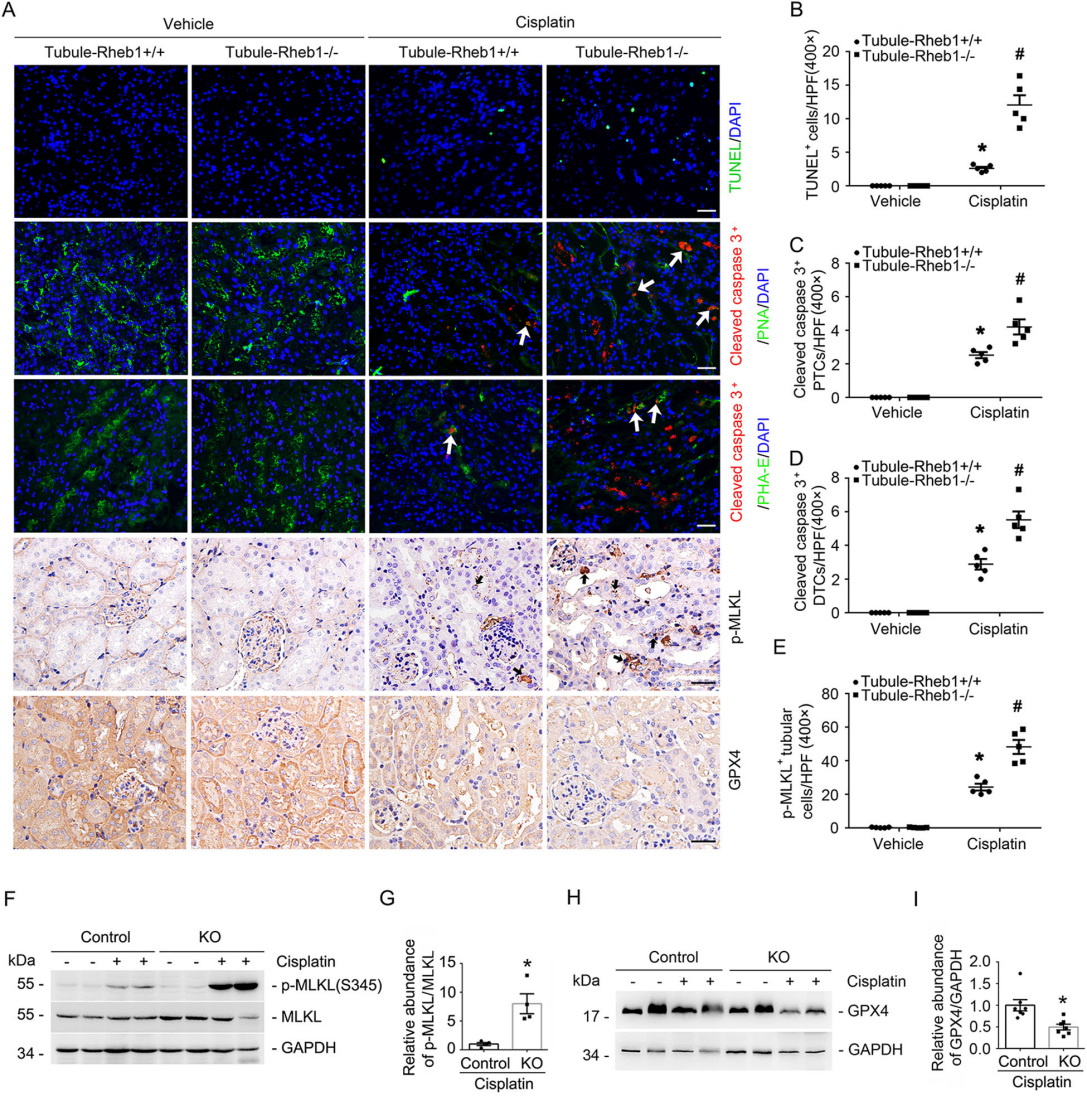
Specific ablation of Rheb1 in tubular cells aggravates tubular cell death in mice after cisplatin injection. **a** Representative micrographs showing TUNEL, anti-cleaved caspase 3, anti-p-MLKL and anti-GPX4 staining as indicated. For anti-cleaved caspase 3 staining, the sections were co-stained with FITC-PHA-E or FITC-PNA to identify proximal or distal tubule, respectively. Arrows indicate cleaved caspase 3-staining positive proximal tubular cells or distal tubular cells. Black arrows indicate anti-p-MLKL staining positive cells. The staining intensity indicate GPX4 abundance. Scale bar = 20 μm. **b** Quantitative analysis of TUNEL-staining positive cells among groups as indicated. *P < 0.05 vs. Vehicle control, *n* = 3–5, #*P* < 0.05 vs. Tubule-Rheb1^+/+^ mice injected with cisplatin, *n* = 5. Each dot represents the average of five HPFs from each mouse. **c**, **d** Quantitative analyses of cleaved caspase 3 staining positive proximal tubular cells (PTCs) and distal tubular cells (DTCs) among groups, *P < 0.05 vs. Vehicle control, *n* = 3–5, #*P* < 0.05 vs. Tubule-Rheb1^+/+^ mice injected with cisplatin, *n* = 5. Each dot represents the average of 6-8 HPFs from each mouse. **e** Quantitative analysis of p-MLKL-staining positive cells among groups as indicated. *P < 0.05 vs. Vehicle control, *n* = 3–5, #*P* < 0.05 vs. Tubule-Rheb1^+/+^ mice injected with cisplatin, *n* = 5. Each dot represents the average of five HPFs from each mouse. **f**–**i** Western blot assay (**f**, **h**) and quantitative analysis (**g**, **i**) showing the abundance of p-MLKL and GPX4 in kidneys from mice injected with cisplatin. **P < 0.01 vs. Tubule-Rheb1^+/+^ mice injected with cisplatin, *n* = 4.

Many studies revealed that apoptosis, necroptosis as well as ferroptosis all contribute to cisplatin-induced tubular cell death and AKI^24–26^. In this study, we firstly used terminal deoxynucleotidyl transferase-mediated dUTP nick-end labeling (TUNEL) staining and anti-cleaved caspase 3 immunofluorescent staining to identify cell apoptosis in the kidney tissues. Few TUNEL- or anti-cleaved caspase 3-staining positive cells was detected in the kidneys from Tubule-Rheb1^+/+^ and Tubule-Rheb1^−/−^ mice without cisplatin treatment. At day 3 after cisplatin injection, compared to those in Tubule-Rheb1^+/+^ mice, the number of TUNEL-staining positive cells was significantly increased in Tubule-Rheb1^−/−^ mice (Fig. 3a, b). To further identify cell apoptosis within the different tubular segments, we co-stained kidney tissues with anti-cleaved caspase 3 antibody and FITC-conjugated proximal tubular cell marker PHA-E or FITC-conjugated distal tubular cell marker PNA, respectively. The quantitative analysis results showed that much more cell apoptosis was detected in both proximal and distal tubule of the knockouts at day 3 after cisplatin injection than those in the control littermates (Fig. 3a, c, d). Then, western blot analysis and immunohistochemical staining for p-MLKL and GPX4 in kidney tissues were used to identify cell necroptosis and ferroptosis, respectively (Fig. 3a, e–i). The number of p-MLKL-staining positive tubular cells was largely increased in Tubule-Rheb1^−/−^ mice compared to their control littermates after cisplatin injection (Fig. 3a, e). Western blot assay confirmed these results (Fig. 3f, g). The expression of GPX4, a suppressor of ferroptosis, was significantly reduced in Tubule-Rheb1^−/−^ mice after cisplatin injection compared to their control littermates after cisplatin injection (Fig. 3h, i). Thus, it is concluded that deletion of Rheb1 in tubular cells promotes cisplatin-induced tubular cell death.

### Specific deletion of Rheb1 in tubular cells aggravates cisplatin-induced tubular cell mitochondrial defect in mice

**Fig. 4:**
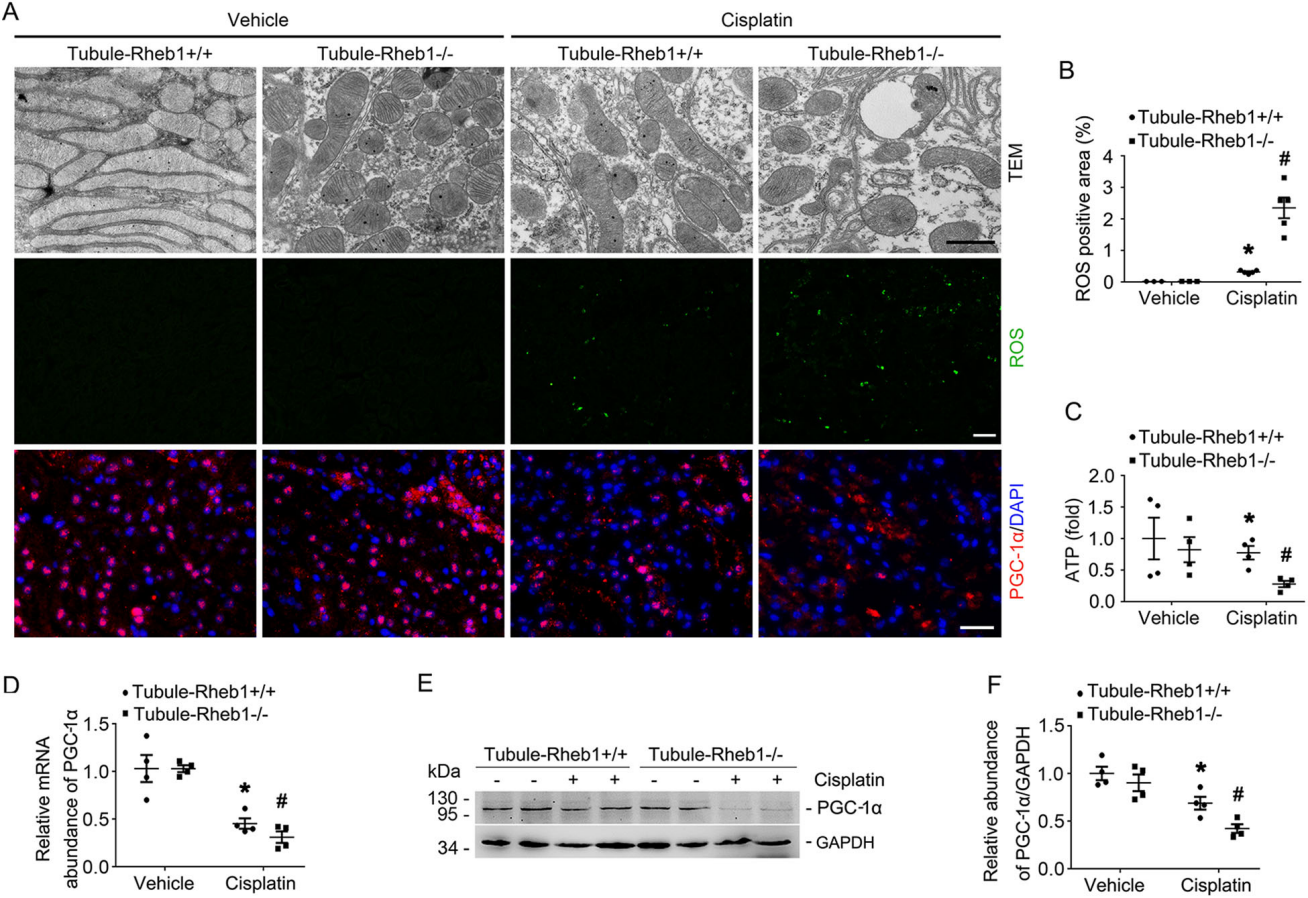
Specific ablation of Rheb1 in tubular cells aggravates PGC-1*α* downregulation and mitochondrial defect in kidney tubule from mice after cisplatin injection. **a** Representative transmission electron micrographs of tubular cells from Tubule-Rheb1^+/+^ and Tubule-Rheb1^−/−^ mice, Scale bar = 2 μm; Representative micrographs showing DCFH-DA staining for ROS (Scale bar = 20 μm) and immunofluorescent staining for PGC-1*α* in kidney tissues (Scale bar = 20 μm). For PGC-1α staining, kidney sections were counterstained with DAPI. **b** Quantitative analysis of DCFH-DA -staining positive area among groups as indicated. Data is presented as the percentage of the staining positive area. *P < 0.05 vs. Vehicle control, *n* = 3–5, #*P* < 0.05 vs. Tubule-Rheb1^+/+^ mice injected with cisplatin, *n* = 5. Each dot represents the average of five HPFs from each mouse. **c** The graph showing the ATP level in kidneys from Tubule-Rheb1^+/+^ and Tubule-Rheb1^−/−^ mice respectively^. *^P < 0.05 vs. Vehicle control, *n* = 4, #P < 0.05 vs. Tubule-Rheb1^+/+^ mice injected with cisplatin, *n* = 4. **d** Real-time q-PCR analysis showing the mRNA abundance for PGC-1*α* in the kidneys. **e**, **f** Western blot assay (**e**) and quantitative analysis (**f**) showing the abundance of PGC-1*α* in the kidneys. *P < 0.05 vs. Vehicle control, #*P* < 0.05 vs. Tubule-Rheb1^+/+^ mice injected with cisplatin, *n* = 4.

Maintenance of mitochondrial homeostasis is essential for keeping tubular cell physiologic function and survival. To evaluate the role of Rheb1 on mitochondrial homeostasis in tubular cells, we first performed transmission electron microscopy (TEM) examination on Tubule-Rheb1^+/+^ and Tubule-Rheb1^−/−^ kidneys with or without cisplatin injection. Without cisplatin injection, Tubule-Rheb1^+/+^ mice showed long and densely packed mitochondria, while Tubule-Rheb1^−/−^ mice showed shortened and mild swollen mitochondria in tubular cells. At day 3 after cisplatin injection, Tubule-Rheb1^+/+^ mice presented shortened and fragmented mitochondria with vacuoles, while the mitochondrial defect in the tubular cells was much severe in Tubule-Rheb1^−/−^ mice (Fig. 4a). We then detected reactive oxygen species (ROS) and ATP level in the kidneys from Tubule-Rheb1^+/+^ and Tubule-Rheb1^−/−^ mice. We used DCFH-DA, a fluorescence probe, to detect the ROS level within kidney tissues and green fluorescence area was calculated. No significant difference was found for ROS and ATP level between Tubule-Rheb1^+/+^ and Tubule-Rheb1^−/−^ kidneys without cisplatin injection. After cisplatin injection, more ROS as well as less ATP were observed in Tubule-Rheb1^−/−^ kidneys compared to those in Tubule-Rheb1^+/+^ kidneys (Fig. 4a–c). To sum up, ablation of Rheb1 may aggravate cisplatin-induced tubular cell mitochondrial defect in mice.

The peroxisome proliferator-activated receptor γ coactivator-1*α* (PGC-1*α*) has been recognized as the main upstream transcriptional regulator of mitochondrial biogenesis and function^27^. In Tubule-Rheb1^+/+^ kidneys, both mRNA and protein abundance of PGC-1*α* were significantly decreased after cisplatin injection. Western blot assay and immunohistochemical staining results showed more reduction of PGC-1*α* expression in Tubule-Rheb1^−/−^ kidneys compared to those in Tubule-Rheb1^+/+^ kidneys after cisplatin injection (Fig. 4a, d–f). Together, these data uncovers that Rheb1 deficiency in tubular cells may aggravate cisplatin-induced mitochondria defect through the downregulation of PGC-1*α* in tubular cells in mice.

### Rheb1 deficiency exacerbates cisplatin-induced tubular cell death in in vitro

**Fig. 5:**
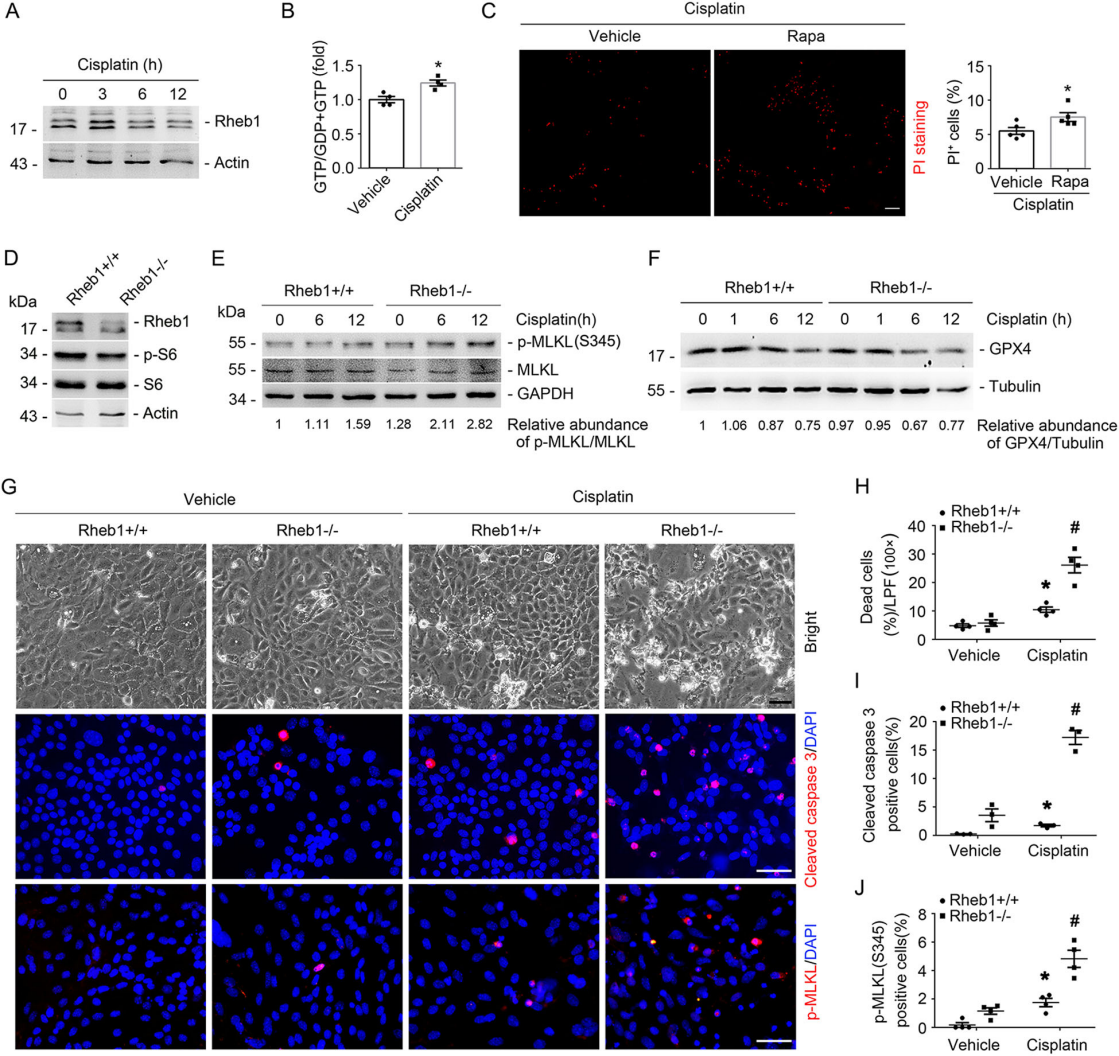
Ablation of Rheb1 in primary cultured tubular cells exacerbates cisplatin-induced cell death. **a** Western blot assay showing the induction of Rheb1 in primary cultured tubular cells incubated with 25 mg/ml cisplatin for 3, 6, and 12 h. **b** GTP loading assay showing the induction of GTP-Rheb1 after cisplatin treatment at 3 h. *P < 0.05 vs. Vehicle control, *n* = 4. **c** PI staining and quantitative analysis of dead cells after cisplatin treatment. Data is presented as the percentage of PI-staining positive cells. *P < 0.05 vs. vehicle control cells, *n* = 5. Scale bar = 20 µm. **d** Western blot assay showing the reduction of Rheb1 and p-S6 abundance in primary cultured tubular cells with Rheb1 ablation. The primary cultured tubular cells from Rheb1^fl/fl^ mice were infected with adenovirus carrying GFP or Cre recombinase gene for 48 h, respectively. **e**, **f** Western blot assay of p-MLKL (**e**) and GPX4 (**f**) in primary cultured tubular cells after cisplatin administration. **g** Representative micrographs showing the cell detachment, anti-cleaved caspase 3 and anti-p-MLKL staining as indicated. Scale bar = 20 µm. **h**–**j** Quantitative analyses of dead cells, cleaved caspase 3-staining positive and p-MLKL-staining positive cells among groups as indicated. Data is presented as the percentage of the staining positive cells. *P < 0.05 vs. vehicle control, #*P* < 0.05 vs. Ad-GFP-infected cells treated with cisplatin, *n* = 3–4.

To investigate the role of Rheb1 in regulating tubular cell survival in in vitro, we firstly tested Rheb1 expression and activity in primary cultured tubular cells after cisplatin treatment. The result of western blot showed that Rheb1 expression were markedly increased after cisplatin stimulation at 3 h. Thereby, we examined active GTP-bound Rheb1 according to our previous publication^21^. The induction of the GTP loading Rheb1 was significantly increased after cisplatin stimulation at 3 hours (Fig. 5a, b). We also treated primary cultured tubular cells with rapamycin to block mTORC1, which was followed by cisplatin treatment. PI staining assay showed blocking mTORC1 could significantly promote cisplatin-induced tubular cell death (Fig. 5c). Then, we infected the primary cultured tubular cells isolated from Rheb1^fl/fl^ mice with adenovirus carrying Cre recombinase (Ad-Cre) to induce Rheb1 gene ablation. The tubular cells infected with adenovirus carrying GFP (Ad-GFP) were considered as control cells. Western blot results showed the obvious reduction of Rheb1 and p-S6 in Ad-Cre-infected tubular cells (Fig. 5d). At 48 h after adenovirus infection, the primary cultured tubular cells were treated with cisplatin for 12 h to induce cell death. Western blot results showed that p-MLKL abundance was largely increased, and GPX4 expression was markedly decreased in Rheb1 deficient cells compared to those in control cells after cisplatin treatment (Fig. 5e, f). The immunofluorescent staining results showed that the number of cleaved caspase 3- and p-MLKL-staining positive cells were both significantly increased in Rheb1 deficient cells compared to those in control cells after cisplatin treatment (Fig. 5g–j). Together, these results suggest that ablating Rheb1 promotes cisplatin-induced cell death in primary cultured tubular cells.

### Rheb1 deficiency aggravates cisplatin-induced mitochondrial defect in primary cultured tubular cells

**Fig. 6:**
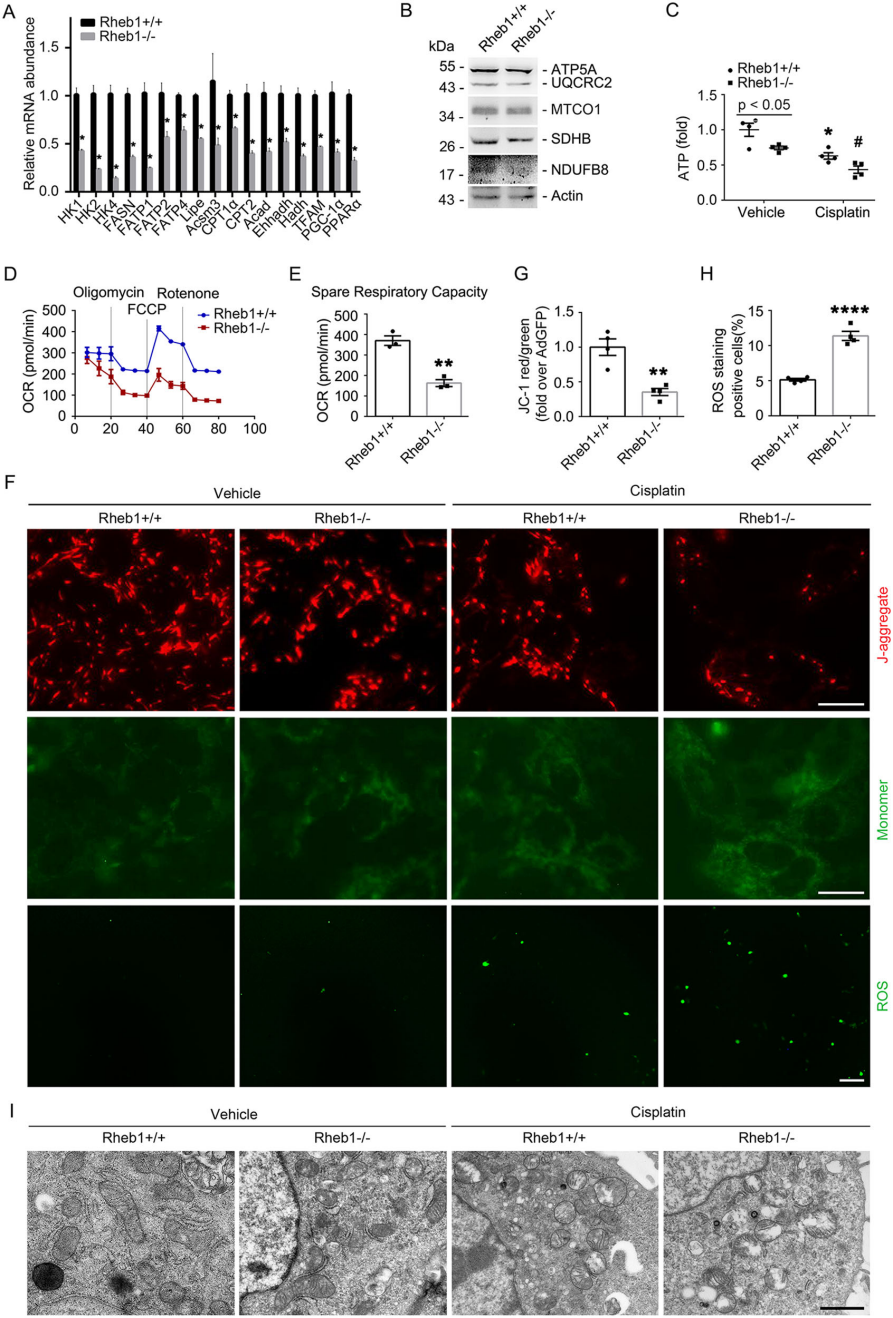
Ablation of Rheb1 in primary cultured tubular cells leads to mitochondrial defect. **a** Real-time qRT-PCR analysis showing the mRNA abundance of mitochondrial related genes for primary cultured tubular cells. **b** Western blot assay showing the abundance of mitochondrial respiratory chain complexes in primary cultured tubular cells. **c** The graph showing the ATP level in primary cultured tubular cells with or without cisplatin treatment. *P < 0.05 vs. Vehicle control, #*P* < 0.05 vs. Ad-GFP-infected cells treated with cisplatin, *n* = 4. **d**, **e** The graph showing the results of OCR assay, **P < 0.01, *n* = 4. **f** Representative images of DCFH-DA staining for ROS (Scale bar = 20 µm) and JC-1 staining (Scale bar = 5 µm). **g**, **h** Quantitative analyses of JC-1 fluorescence intensity (red/green) and DCFH-DA -staining positive cells after cisplatin treatment. Data is presented as the percentage of the staining positive cells. *P < 0.05 vs. vehicle control, #*P* < 0.05 vs. Ad-GFP-infected cells treated with cisplatin, *n* = 4. **i** Representative transmission electron micrographs of the primary cultured tubular cells infected with Ad-Cre and Ad-GFP with or without cisplatin treatment as indicated. *P < 0.05 vs. vehicle control. (Scale bar = 2 µm). HK1, 2, and 4: hexokinase 1, 2, and 4; FASN: fatty acid synthase; FATP1, 2, and 4: fatty acid transport protein 1, 2, and 4; HSL or Lipe: hormone-sensitive lipase; Acsm3: acyl-CoA synthetase medium-chain family member 3; Cpt1*α* and 2: carnitine palmitoyl transferase 1*α* and 2; Acad acyl-Coenzyme A dehydrogenase; Ehhadh enoyl-coenzyme A hydratase/3-hydroxyacyl-coenzyme A dehydrogenase; Hadh hydroxyacyl-Coenzyme A dehydrogenase; TFAM mitochondrial transcription factor A; PGC-1*α*: peroxisome proliferator-activated receptor γ coactivator-1*α*; PPAR*α*: peroxisome proliferators-activated receptor *α*.

Compared to the control cells, the mRNA abundance of some mitochondria-related genes, the abundance of mitochondrial respiratory chain complex I, II and III, ATP production and oxygen consumption rate (OCR) were all significantly decreased in Rheb1 deficient tubular cells under normal cultural condition (Fig. 6a–e). The mitochondrial membrane potential level was markedly reduced, and the ROS level was largely increased in Rheb1 deficient tubular cells compared to the control cells after cisplatin treatment (Fig. 6f–h). Rheb1 deficient tubular cells showed more severe mitochondrial crista lesion and fragmentation compared to control cells after cisplatin treatment (Fig. 6i). Together, these results reveal that ablating Rheb1 aggravates cisplatin-induced mitochondrial defect in cultured tubular cells.

### Haploinsufficiency of Tsc1 in tubular cells prevents cisplatin-induced tubular cell death and AKI

**Fig. 7:**
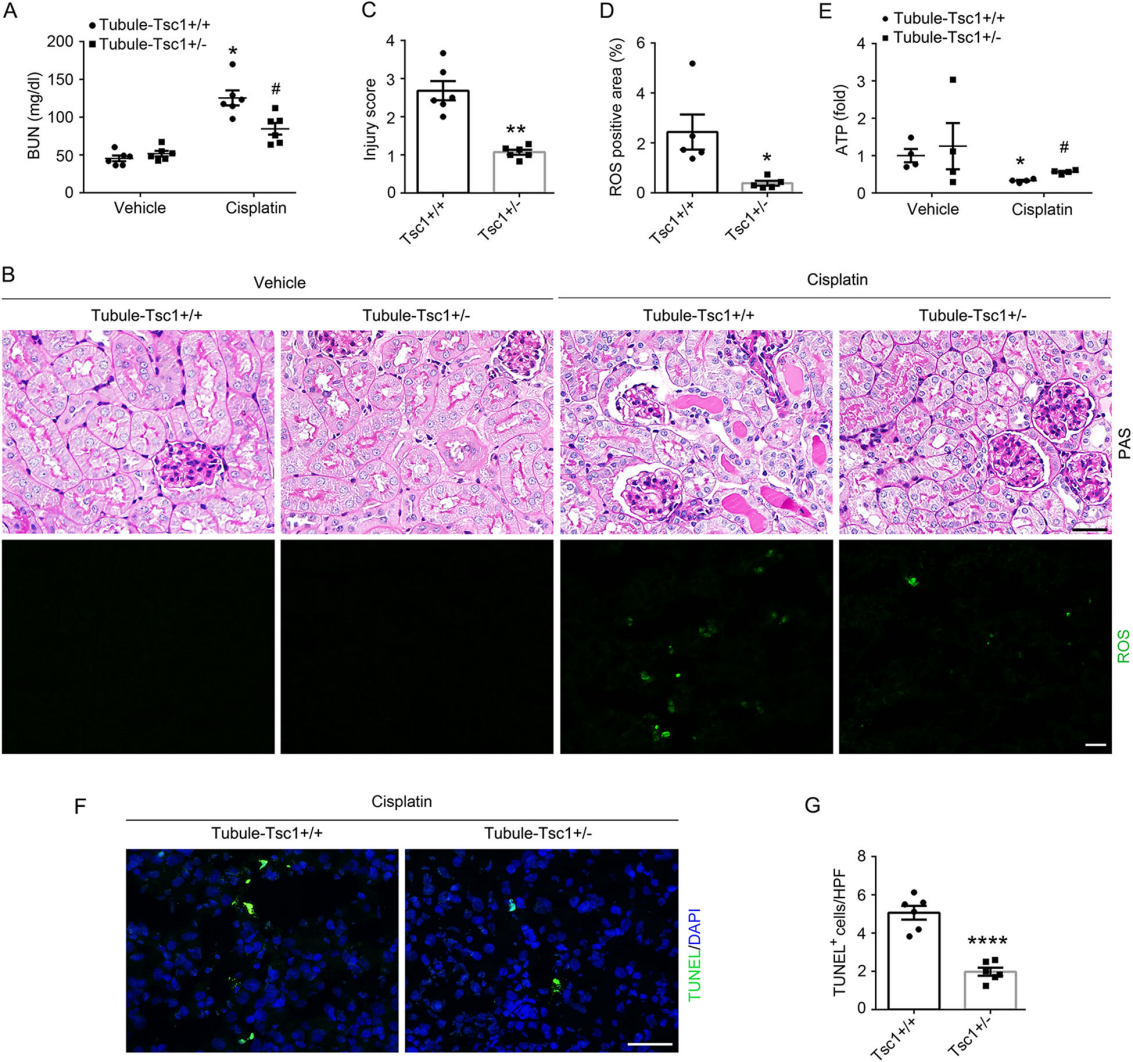
Tubular cell-specific genetic activation of mTORC1 signaling in mice prevents cisplatin-induced tubular cell death and AKI. **a** The graph showing blood urea nitrogen (BUN) level in Tubule-Tsc1^+/−^ and Tubule-Tsc1^+/+^ mice. *P < 0.05 vs. Vehicle control, #*P* < 0.05 vs. Tubule-Tsc1^+/+^ mice injected with cisplatin, *n* = 6. **b** Kidney histology as shown by periodic acid-Schiff staining and representative micrographs of DCFH-DA staining for ROS. Scale bar = 20 µm. **c** Injury scores. **P < 0.01 vs. Tubule-Tsc1^+/+^ mice injected with cisplatin, *n* = 6. **d** Quantitative analysis of DCFH-DA-positive area among groups as indicated. Data is presented as the percentage of the staining positive area. *P < 0.05 vs. Tubule-Tsc1^+/+^ mice injected with cisplatin, *n* = 5. Each dot represents the average of five LPFs from each mouse. **e** The graph showing the ATP level in kidney tissues of Tubule-Tsc1^+/+^ and Tubule-Tsc1^+/−^ mice. *P < 0.05 vs. Vehicle control, *n* = 4, #*P* < 0.05 vs. Tubule-Tsc1^+/+^ mice injected with cisplatin, *n* = 4. **f** Representative micrographs showing TUNEL staining for apoptotic cells. Scale bar = 20 µm. **g** Quantitative analysis of TUNEL-staining positive cells among groups as indicated. Data is presented as the number of TUNEL-staining positive cells per field (×400). ****P < 0.0001 vs. Tubule-Tsc1^+/+^ mice injected with cisplatin, *n* = 6. Each dot represents the average of five HPFs from each mouse.

Tsc1/2 is the main negative regulator of Rheb1. Complete ablation of Tsc1 in tubular cells leads to robust mTORC1 activation and kidney cystic disease. Therefore, in this study, we generated a mouse model with only one allele of Tsc1 gene was deleted in tubular cells (Tubule-Tsc1+/−, Supplementary Fig. 2A, B). Tubule-Tsc1^+/−^ mice were born normal. No significant difference was observed in body weight, kidney weight, kidney/body weight ratio, kidney histology and BUN and urinary NAG level between Tubule-Tsc1^+/+^ and Tubule-Tsc1^+/−^ mice within 2 months after birth (Supplementary Fig. 2C–H). The mice were injected with cisplatin to induce AKI. At day 3 after injection, less BUN level, morphological injury or ROS production, and more ATP production were observed in Tubule-Tsc1^+/−^ mice compared to those in Tubule-Tsc1^+/+^ mice (Fig. 7a–e). TUNEL staining showed that the number of apoptotic cells in the kidneys was significantly decreased in Tubule-Tsc1^+/−^ mice compared to those in Tubule-Tsc1^+/+^ mice after cisplatin injection (Fig. 7f, g).

**Fig. 8:**
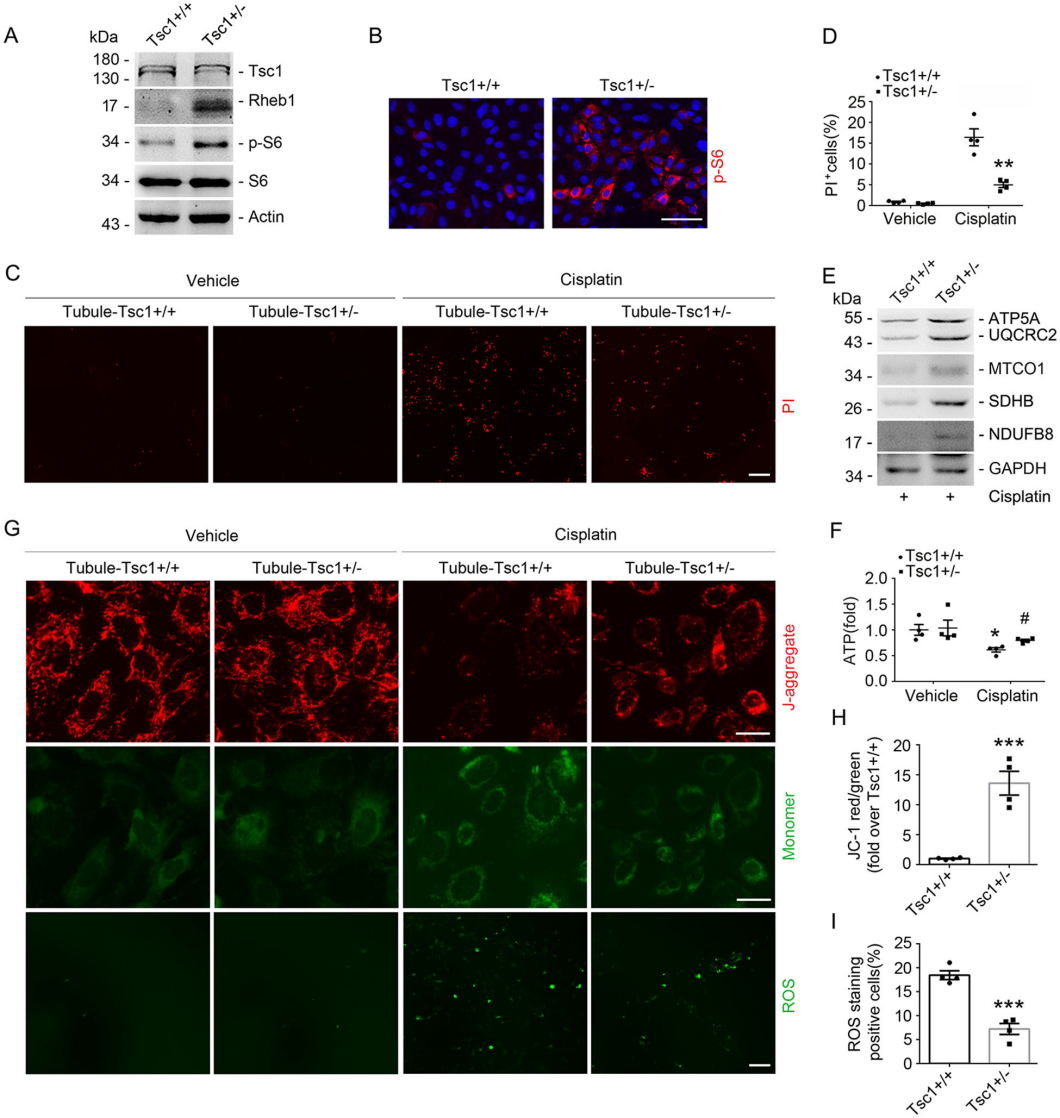
Haploinsufficiency of Tsc1 in primary cultured tubular cells facilitates Rheb1 signaling activation and prevents cisplatin-induced cell death and mitochondrial defect. **a** Western blot assay showing the abundance of Tsc1, Rheb1, p-S6, and S6 in primary cultured tubular cells. **b** Representative immunofluorescent staining images for p-S6 in primary cultured tubular Tsc1^+/+^ and Tsc1^+/−^ cells. Scale bar = 20 µm. **c** PI staining and **d** quantitative analysis of dead cells. Data is presented as the percentage of PI-staining positive cells. **P < 0.01 vs. Tubule-Tsc1^+/+^ cells treated with cisplatin, *n* = 4. Scale bar = 20 µm. **e** Western blot assay showing the abundance of mitochondrial respiratory chain complexes in primary cultured tubular cells. **f** The graph showing the ATP level in primary cultured tubular cells. *P < 0.05 vs. Vehicle control, #*P* < 0.05 vs. Tubule-Tsc1^+/+^ cells treated with cisplatin, *n* = 4. **g** Representative microscopy images of DCFH-DA staining for ROS (Scale bar = 20 µm) and JC-1 staining (Scale bar = 5 µm). **h**, **i** Quantitative analyses of JC-1 fluorescence intensity (red/green) and DCFH-DA-staining positive cells after cisplatin treatment, data is presented as the percentage of the staining positive cells, ***P < 0.001 vs. Tubule-Tsc1^+/+^ cells treated with cisplatin, *n* = 4.

In in vitro, primary cultured tubular cells from Tsc1^fl/wt^ mice were infected with Ad-Cre for 48 h. The Tsc1 abundance was largely decreased and the abundance of Rheb1 and p-S6 were obviously increased in Tubule-Tsc1^+/−^ cells compared to those in the control cells (Fig. 8a, b). PI staining showed that less dead cells were detected in Tubule-Tsc1^+/−^ cells than those in Tubule-Tsc1^+/+^ cells after cisplatin treatment (Fig. 8c, d). Compared to those in Tubule-Tsc1^+/+^ cells, the abundance of mitochondrial respiratory chain complexes was largely elevated in Tubule-Tsc1^+/−^ cells after cisplatin treatment (Fig. 8e). Additionally, mitochondrial defect exhibited as reduced ATP production, increased ROS and decreased mitochondrial membrane potential were alleviated in Tubule-Tsc1^+/−^ cells compared to those in Tubule-Tsc1^+/+^ cells after cisplatin treatment (Fig. 8f–i). Therefore, these results demonstrate that haploinsufficiency of Tsc1 protects against cisplatin-induced mitochondrial defect and tubular cell death.

## Discussion

In this study, by employing mouse models with tubular cell-specific Rheb1 deletion and tubular cell-specific Tsc1 haploinsufficiency, we demonstrated that Rheb1 signaling activation in the kidney tubule protects against tubular cell death and AKI. In addition, we found that Rheb1 may prevent cisplatin-induced tubular cell death via maintaining mitochondrial homeostasis. This study deciphers the mechanism for Rheb1 signaling in regulating tubular cell survival and AKI.

Rheb1, a monomeric protein belonging to Ras superfamily of small GTPases, is highly conserved during evolution, which functions as a molecular switch in various cellular functions^13^. Similar to the other members of the Ras superfamily of small GTPases, the activity of Rheb1 is controlled by its guanine nucleotide binding states. Binding with GTP is an active state of Rheb1 that activates downstream effectors, while it becomes inactive following hydrolysis of the bound GTP to GDP^28^. Grahammer et al. showed tubular cell-specific raptor deletion leads to defective concentrating mechanisms and polyuria^29^. In this study, there was no obviously phenotypic alteration in mice with tubular cell ablation of Rheb1 within two months of birth, suggesting Rheb1 may be dispensable for the physiologic function of tubular cells.

Accumulated studies revealed that Rheb1 may suppress cell death in many cell types including retinal ganglion cells, HeLa cells, colorectal cancer cells and acute myeloid leukemia (AML) cells through different mechanisms^16,20,30–32^. On the contrary, the other studies showed that Rheb1 activation enhances cell apoptosis in HeLa cells and cardiomyocytes^31,33^. Thus, it seems that Rheb1 regulates cell survival through different mechanisms and showing a context-dependent manner. Mice with cisplatin-induced nephrotoxicity may be manifested as renal tubular cell injury and death in all segments of the tubule^34,35^. In Ksp1.3/Cre transgenic mice, Cre recombinase is expressed in the collecting ducts, loops of Henle, distal tubules, as well as proximal tubule with different percentile^36^. Previous studies utilized this strain to generate mouse models with target gene ablation in the tubule and explore the function of those gene products in cisplatin nephrotoxicity^37,38^. Therefore, in this study, we employed this mice to generate mouse model with tubular Rheb1 deletion and investigate the role of tubular Rheb1 in regulating cisplatin nephrotoxicity. We found that Rheb1 deletion promoted cisplatin-induced tubular cell death including apoptosis, necroptosis and ferroptosis based on the evidences from both in in vivo and in vitro studies. The routes of cell death in this study were supported by the effects of the necroptosis inhibitor (Nec-1) and ferroptosis inhibitor (Fer1) in protecting against the cisplatin-induced tubular cell death and AKI in mice^26,39^. In mouse model with tubular cell-specific ablation of Rheb1, more severe tubular cell death and AKI were observed after cisplatin injection. Ablation of Rheb1 in primary cultured tubular cells promoted cisplatin-induced tubular cell death. As we know, Tsc1 acts as a GTPase activating protein which stimulates GTP hydrolysis of Rheb1 and inhibits its activation^40,41^. Previous studies showed that mice with the loss of either Tsc1 or Tsc2 are prone to develop some disorders such as hyperkalemia^42^, renal tumor with hemangiosarcoma^43^, and polycystic kidney disease in mice^44^. Zhou et al. uncovered that complete loss of Tsc1 in renal tubular cells causes polycystic kidney disease by activating mTORC1 pathway^45^. Due to these obvious disorder phenotypes after Tsc1 or Tsc2 complete deletion, we generated mouse model with tubular cell haploinsufficiency of Tsc1 gene. The results showed that haploinsufficiency of Tsc1 in tubular cells led to Rheb1 signaling activation and protected against cisplatin-induced AKI, suggesting that activation of Rheb1 is sufficient for preventing cisplatin-induced tubular cell death and AKI.

Kidney tubular cells require a large amount of ATP to accomplish transcellular transport and tubular reabsorption^46,47^. Mitochondrial dysfunction results in less ATP production, and alters the cellular function and structure^47^. Decreased ATP production, the disruption of mitochondrial cristae, increased ROS production, and the release of cytochrome c are observed in AKI^48–50^. Rheb1 may be localized on mitochondria^51^. Bcl-2 and Bcl-X_L_ are two membrane proteins that reside on mitochondria. FKBP38, a member of the FK506-binding protein family, inhibits apoptosis via anchoring Bcl-2 and Bcl-X_L_ to mitochondria^52^. Rheb1 may restrain apoptosis by regulating the interaction of FKBP38 with Bcl-2 and Bcl-X_L_^32^. Melser et al. found that Rheb1 induces mitophagy, inhibits ROS-induced damage, and increases basal and maximal mitochondrial respiration^16^. In this study, we found that Rheb1-deficient mice yield shortened and fragmented mitochondria with irregular and disordered shape, and vacuoles, indicating Rheb1 is important in maintaining the structure and function of mitochondria, which is in line with the report from Melser et al.^16^. PGC-1*α* is a primary regulator of mitochondrial biogenesis and function and highly expressed in the kidney^53,54^. Increased cellular PGC-1*α* level promotes mitochondrial biogenesis in many types of cell^55,56^. In this study, Rheb1 deletion downregulated PGC-1*α* expression in tubular cells. Thus, Rheb1 may keep mitochondrial homeostasis through upregulating PGC-1*α* expression. However, the underlying mechanism of Rheb1 in regulating PGC-1*α* expression needs more investigation.

In addition to tubular cells, our published studies reported that Rheb1 activation in fibroblasts may protect against tubular cell death and AKI through stimulating HGF production^22^. However, constitutive activation of Rheb1 in kidney fibroblasts may lead to interstitial fibrosis in mice^21^. Therefore, targeting Rheb1 for patients with chronic kidney disease should be cautious.

To sum up, this study shows that tubular Rheb1 alleviates cisplatin-induced AKI via maintaining mitochondrial homeostasis. Activation of Rheb1 may provide a new therapeutic strategy for AKI.

## Supplementary information

Supplemental Methods

Supplemental Figure 1

Supplemental Figure 2
