# Supplementary material for: Rheb1 protects against cisplatin-induced tubular cell death and acute kidney injury via maintaining mitochondrial homeostasis
Source: Cell Death Dis. 2020 May 13;11(5):364. doi: 10.1038/s41419-020-2539-4 (PMC7221100; doi:10.1038/s41419-020-2539-4)
Supplement: Supplementary file 1 — Supplemental Methods [file 41419_2020_2539_MOESM1_ESM.docx]

**Supplemental Methods:**

**Assessment of Kidney Function**

BUN in serum was measured with the QuantiChrom Urea Assay kit (cat: DIUR-500, Hayward, U.S.A) according to the manufacturer’s instructions. Urinary NAG was detected by using the NAG assay kit (Nanjing Jiancheng Bioengineering, Nanjing, China).

**Histology and immunohistochemistry**

Human kidney specimens diagnosed with AKI by independent pathologists were obtained from diagnostic renal biopsies performed at the second affiliated hospital of Nanjing Medical University. The adjacent kidney tissues of kidney tumor patients from the second affiliated hospital of Nanjing Medical University were used as controls. Of these patients, mean age +/- STDEV was 48.33+/- 10.46 years for AKI patients (4 males and 2 females), and 57.33+/- 8.066 years for controls (3 males and 3 females). The Institutional Review Board at the second affiliated hospital approved all studies involving human tissues.

Paraffin-embedded kidney sections (3-μm thickness) were stained with periodic acid-Schiff. A semiquantitative scoring method was used to define kidney injury by the loss of brush border, tubular cell necrosis and cellular casts. Score 0 represents injury area less than 10%, whereas score 1, 2, 3, and 4 represent the injury involving 10-25%, 25-50%, 50-75% and >75% of the kidney tissue area, respectively. At least five randomly selected fields under ×400 microscope were evaluated for each mouse, and an average score was calculated.

For immunohistochemical staining, paraffin-embedded kidney tissue sections were deparaffinized and hydrated. Heat-induced epitope retrieval (HIER) was employed. Briefly, a steamer or water bath with a staining dish containing sodium citrate buffer or citrate buffer was preheated until the temperature reached 95-100°C. Slides were immersed in the staining dish. The lid was placed loosely on the staining dish, and the slides were incubated for 20-40 min. The staining dish was then removed at room temperature, and the slides were allowed to cool for 20 min before the normal staining procedure commenced. Endogenous peroxidase activity was quenched by 3% H2O2. Tissue sections were then blocked with 10% normal donkey serum, followed by incubation with anti-p-S6 (cat: 4858, Cell Signaling Technology), anti-Rheb1 (cat: ab25873, abcam) and anti-p-MLKL (cat: ab196436, abcam) overnight at 4°C. After incubation with secondary antibody for 1 h, sections were incubated with ABC reagents for 1 h at room temperature before subjected to substrate 3-amino-9-ethylcarbazole for staining (Vector Laboratories, Burlin-game, CA). Slides were viewed with a Nikon Eclipse 80i Epifluorescence microscope equipped with a digital camera (DS-Ri1, Nikon).

**Immunofluorescent staining**

Kidneys were frozen in Optimum Cutting Temperature (O.C.T.) compound and sectioned at 3 μm thickness (Leica Kryostat). The sections were fixed with 4% paraformaldehyde for 15 min, followed by permeabilization with 0.5% Triton X-100 in PBS for 5 min at room temperature, and then blocked with 2% donkey serum for 60 min. The sections were then immunostained with the following antibodies: anti-Rheb1 (cat: ab25873, abcam), anti-cleaved caspase 3 (cat: 9664, Cell Signaling Technology), anti-PGC1α (cat: ab54481, abcam), anti-Ly6b (cat: MCA771G, AbD Serotec, Raleigh, NC) and anti-F4/80 (catalog no. 14-4801, eBioscience, San Diego, CA), respectively. To determine Rheb1 expression in different tubule segments, the sections were co-stained with FITC-PHA-E (cat: P3370, US Biological) or FITC-PNA (cat: L7381, Sigma, St Louis, MO) to identify proximal or distal tubule, respectively.

Primary cultured tubular epithelial cells seeded on coverslips were fixed with cold methanol/acetone (1:1) for 10 min at -20 °C. After three extensive washings with 1×PBS, cells were treated with 1% Triton X-100 for 5 minutes, blocked with 2% normal donkey serum in 1×PBS buffer for 40 minutes at room temperature, and incubated with the following antibodies: anti-p-MLKL (cat: ab196436, abcam), anti-cleaved caspase 3 (cat: 9664, Cell Signaling Technology) followed by staining with FITC or tetramethylrhodamine-conjugated secondary antibodies. Cells were also stained with 49,6-diamidino-2-phenylindole to visualize the nuclei. Slides were viewed with a Nikon Eclipse 80i Epi-fluorescence microscope equipped with a digital camera.

**TUNEL staining**

The primary cultured tubular cells isolated from Rheb1^fl/fl^ mice were seeded in 12-well plate and then infected with adenovirus carrying Cre recombinase (Ad-Cre) or GFP (Ad-GFP). At 48 h after adenovirus infection, the primary cultured tubular cells were treated with cisplatin for 12 h to induce cell death. The kidney sections with 5 μm thickness from Tubule-Rheb1^+/+^ and Tubule-Rheb1^-/-^ mice with cisplatin injection for 3 days were prepared for determination of apoptotic cells. For determination of apoptotic cells, terminal deoxynucleotidyl transferase-mediated dUTP nick-end labeling staining was used with Apoptosis Detection System (Promega, Madison, WI). Slides were viewed with a Nikon Eclipse 80i Epi-fluorescence microscope equipped with a digital camera. Five fields were randomly selected from each slide for quantitative analysis of TUNEL-staining positive cells among groups. The average of TUNEL-staining positive cells from five fields represents the number of TUNEL-staining positive cells in each slide.

**Transmission electron microscopy (TEM)**

The kidney sections were fixed in 3.7% glutaraldehyde in PBS buffer. After rinsing and post-fixing in 1% osmium tetroxide, samples were embedded in 10% gelatin, fixed and cut into several blocks (< 1 mm^3^). After dehydrating in increasing concentrations of alcohol and infiltrated with increasing concentrations of Quetol-812 epoxy resin mixed with propylene oxide, samples were embedded in pure, fresh Quetol-812 epoxy resin and polymerized. Ultrathin sections (100 nm) were cut using a Leica UC6 ultra-microtome and post-stained with uranyl acetate for 10 min and with lead citrate for 5 min at room temperature before observation in transmission electron microscope (JEOL JEM-1010, Tokyo, Japan).

After treatment, primary cultured tubular epithelial cells were ﬁxed with 2.5% glutaraldehyde/1.2% acrolein in ﬁxative buffer (0.1 mol/l cacodylate, 0.1 mol/l sucrose, pH 7.4) and 1% osmium tetroxide. Ultrathin sections were stained with uranyl acetate for examination under an electron microscope (JEOL JEM-1010, Tokyo, Japan).

**ATP Assay**

Primary cultured tubular cells isolated from Rheb1^fl/fl^ mice were infected adenovirus carrying Cre recombinase (Ad-Cre) for 48 hours to induce Rheb1 gene ablation. Cells were harvested by lysis buffer (Beyotime, P0013) after cisplatin treatment for 12 hours. The kidneys weighed 20mg from Tubule-Rheb1^+/+^ and Tubule-Rheb1^-/-^ mice with cisplatin injectin for 3 days were also lysed in buffer (Beyotime, P0013). Then the ATP concentration was quantitatively determined using an ATP determination kit (Beyotime, Nanjing, China) according to the manufacturer’s protocol. ATP content was expressed as nmol/mg protein.

**Mitochondrial membrane potential measurement**

Primary cultured tubular cells isolated from Rheb1^fl/fl^ mice were infected adenovirus carrying Cre recombinase (Ad-Cre) for 48 hours to induce Rheb1 gene ablation. The primary cultured tubular cells treated with cisplatin for 10 hours were tested mitochondrial membrane potential by exposing to JC-1 (T3168, Molecular Probes, Invitrogen, Waltham, MA, USA). Briefly, cells were washed twice with PBS, and JC-1 was added to each well with a final concentration of 300 nM for 20 min. Change of membrane potential was indicated by a fluorescence shift from red to green. Five HPF fields were randomly selected from each group for quantitative analysis of the ratio of J-aggregate to Monomer fluorescent densities among groups. The ratio of J-aggregate to Monomer fluorescent densities to the selected field for five randomly selected fields from each group were analyzed with Image Pro Plus 6.0, and an average ratio of J-aggregate to Monomer fluorescent densities for each group was calculated.

**ROS Assay**

ROS was measured by exposing cells and kidney sections to DCFH-DA (S0033-1 10 μM for 20 min, 37 °C, Fluorescence Probe, Beyotime Biotechnology, China). DCFH-DA was hydrolyzed into non-fluorescent DCFH. Next, DCFH was oxidized by ROS into fluorescent DCF which reflected the level of intracellular ROS. Five fields were randomly selected from each group for quantitative analysis of ROS-staining positive cells among groups. The average of ROS-staining positive cells from five fields represents the number of ROS-staining positive cells in each group.

**Mitochondrial stress assay**

An assay using the Seahorse XF-96 Extracellular Flux Analyzer (Sea-horse Bioscience, Copenhagen, Denmark) was performed to measure the OCR. Briefly, primary cultured tubular epithelial cells were initially cultured in dish. On the time of the experiment, cell media was replaced by Seahorse assay media and then cells were infected with adenovirus carrying Cre recombinase gene to generate tubular cell Rheb1 ablation before assessing basal OCR. Inhibitors were prepared in the same medium and the injection ports of the sensors were filled. Thirty minutes before the experiment, the sensor was placed into the XF-96 instrument and calibration was initiated. After calibration, the basal oxygen consumption was recorded for 20 minutes and OCR measurements were performed over time upon the successive addition of the mitochondrial inhibitors: oligomycin (1 mM), which blocks the proton channel of the portion of ATP synthase (complex V) and thus inhibits ATP synthesis used to determine ATP-synthesis coupling efficiency; FCCP (1 mM) was used to calculate the spare respiratory capacity; and finally, a mixture of rotenone (0.5 mM) and antimycin A (0.5 mM), inhibiting complex I and complex III respectively, was used to assess the consumption of oxygen of nonmitochondrial origin.

**Measurement of Rheb1 Activation in** **Primary Cultured Tubular Cells**

Primary cultured tubular cells were treated with cisplatin for 3 hours as indicated. Cells were harvested after being washed two times by cold PBS, and extracted in an ice-cold 50 mM HEPES-based buffer (PH 7.4) containing 10 mM MgCl_2_, 150 mM NaCl, 1% Nonidet P-40, 0.5 mM phenylmethylsulfonyl fluoride, protease inhibitor and phosphatase inhibitors. After gentle shaking for 10 min at 4°C the lysed cells were centrifuged at 11,000g for 10 min. The total Rheb1 in the supernatants was precipitated with 3 μg of anti-Rheb1 antibody. After shaking for 16 hours at 4°C, protein G-agarose as well as NaCl, sodium dodecyl sulfate (SDS), and deoxycholate were added into samples with final concentrations of 500 mM, 0.05%, and 0.5%, respectively. The samples were shaken gently for 1 h at 4 °C and then the immunoprecipitates were washed four times with cold buffer containing 50 mM Tris HCl (pH 7.4), 10 mM MgCl2, 500 mM NaCl, 0.1% Triton X-100, 0.005% SDS and two times with 20 mM Tris phosphate (pH 7.8). After the last wash the immunoprecipitates were resuspended in 30μl of 5 mM Trisphosphate (pH 7.4), 2 mM dithiothreitol (DTT), 2 mM EDTA and then heated at 100°C for 3 min to elute GTP and GDP bound to the precipitated Rheb1. The samples were cooled on ice and centrifuged at 11,000g for 5 min. GTP was converted to ATP using NDP kinase and ADP with the resulting ATP measured in the luciferase/luciferin system with the ATP Bioluminescent Assay Kit (product number: FL-AA; Sigma-Aldrich). The sum of GTP plus GDP was measured by converting GDP to GTP using pyruvate kinase and phosphoenolpyruvate and then the total GTP, representing the sum of GDP plus GTP, was measured as described above. The reaction mixture was incubated for 30 min at 30 °C. One reaction mixture was contained in a final volume of 15μl of 50 mM glycine (pH 7.8), 10 mM dithiothreitol, 8 mM MgSO4, 50 mM phosphoenolpyruvate, 3 milliunits of pyruvatekinase, and 5 μl of sample. The other was contained in a final volume of 15μl of 50 mM glycine (pH 7.8), 10 mM dithiothreitol, 8 mM MgSO4, 50 mM phosphoenolpyruvate, 3 milliunits of pyruvatekinase, 0.7 μM ADP, 2.5 milliunits of NDP kinase, and 5 μl of sample. The result was presented as the ratio of GTP to GTP plus GDP.

**Western blot analysis**

Primary cultured tubular cells were harvested with 1×SDS sample buffer. The kidneys were lysed with RIPA solution containing 1% NP40, 0.1% SDS, 100 mg/ml PMSF, 1% phosphatase I and II inhibitor cocktail, and 1% protease inhibitor cocktail (Sigma, St Louis, MO) on ice for 30 min. The supernatants were collected after centrifugation at 16,000 g at 4 °C for 30 min. Protein concentration was determined by the bicinchoninic acid protein assay (BCA Kit; Pierce Thermo-Scientific, Rockford, IL) according to the manufacturer’s instruction. An equal amount of protein was loaded into 10% or 15% SDS-PAGE and transferred onto polyvinylidene difluoride membranes. The primary antibodies were as the following: anti-p-MLKL (cat: ab196436, abcam), anti-MLKL (cat: 37705, Cell Signaling Technology), anti-p-S6 (cat: 4858, Cell Signaling Technology), anti-S6 (cat: 2217, Cell Signaling Technology), anti-Rheb1 (cat: ab25873, abcam), anti-Tsc1 (cat: 4906, Cell Signaling Technology), anti-PGC-1α (cat: ab54481, abcam), anti-β-actin (cat: sc-47778, Santa Cruz Biotechnology), and anti-GAPDH (cat: FL-335, Santa Cruz Biotechnology, Dallas, TX). Quantification was performed by measuring the signal intensity with the aid of National Institutes of Health Image J software package.

**Real-time qRT-PCR**

Total RNA was extracted with Trizol reagent (Invitrogen) according to the manufacturer’s instructions. cDNA was synthesized with 1 μg of total RNA, ReverTra Ace (Vazyme, Nanjing, China), and oligo (dT) 12-18 primers. Real-time qRT-PCR assay was used to quantitate relative mRNA abundance with Light Cycler 96 Systems (Roche). The relative amount of mRNA to internal control was calculated using the equation 2ΔCT, in which ΔCT = CT^gene^ - CT^control^.
