# Supplementary figures and images for: Rheb1 protects against cisplatin-induced tubular cell death and acute kidney injury via maintaining mitochondrial homeostasis
Source: Cell Death Dis. 2020 May 13;11(5):364. doi: 10.1038/s41419-020-2539-4 (PMC7221100; doi:10.1038/s41419-020-2539-4)

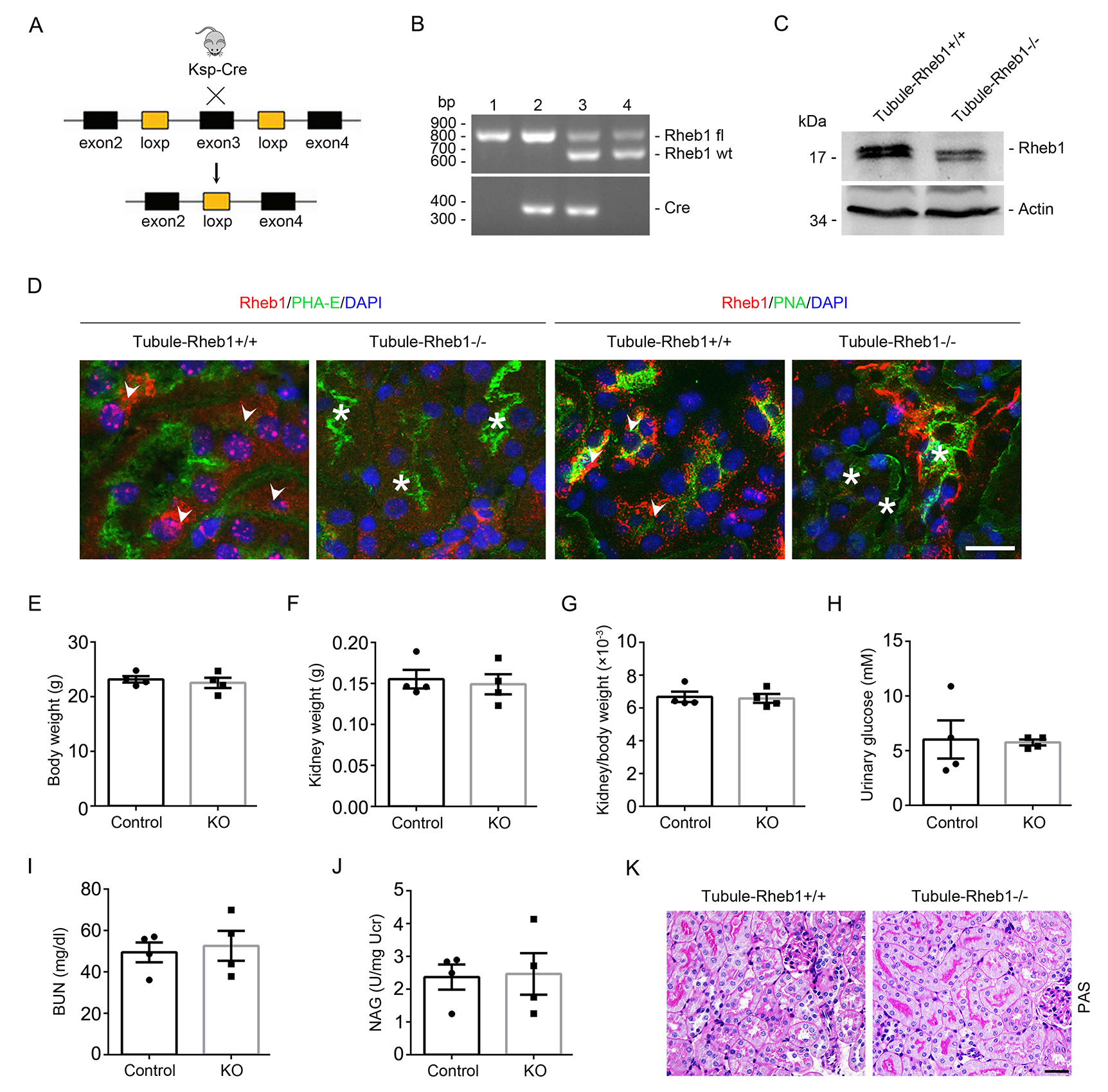

Supplement: Supplementary file 2 — Supplemental Figure 1 [file 41419_2020_2539_MOESM2_ESM.tif]

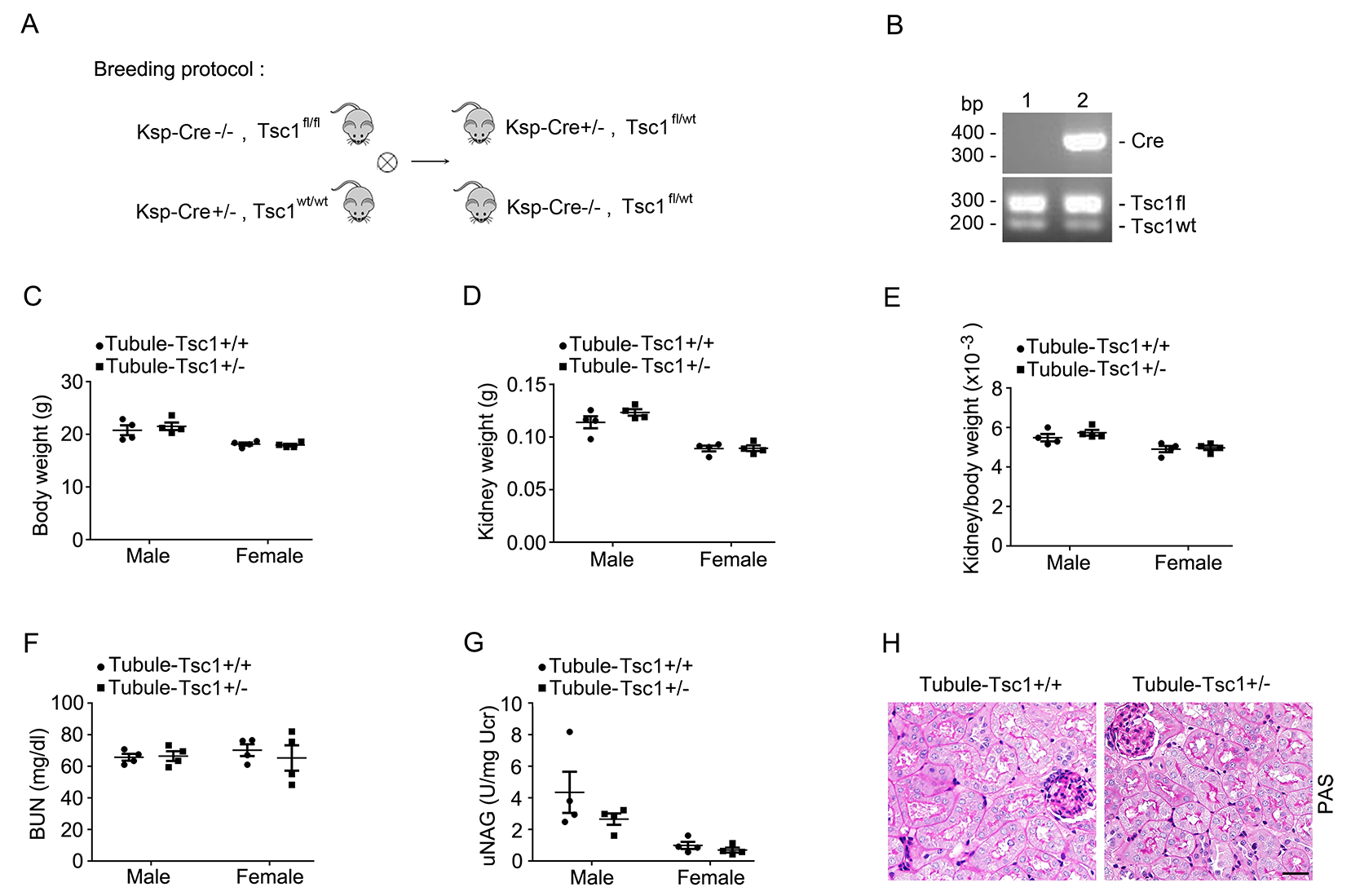

Supplement: Supplementary file 3 — Supplemental Figure 2 [file 41419_2020_2539_MOESM3_ESM.tif]
